# How We View Our Jobs and Our Clients: A Quantitative Study of Rejection Sensitivity in Trauma-Informed Care

**DOI:** 10.3390/bs15121733

**Published:** 2025-12-15

**Authors:** Xiwei Huang, Emily A. Bosk, Alicia Mendez, Tareq Hardan, Gina Everett, Michael J. MacKenzie

**Affiliations:** 1 Institute of Sociology, Sichuan Academy of Social Sciences, Chengdu 610072, China; 2 School of Social Work, McGill University, Montreal, QC H3A 0G4, Canada; 3 School of Social Work, Rutgers University, New Brunswick, NJ 08901, USA; emily.bosk@ssw.rutgers.edu; 4 School of Social Work, Bridgewater State University, Bridgewater, MA 02324, USA; 5 School of Social Work, University of Northern British Columbia, Prince George, BC V2N 4Z9, Canada; tareq.hardan@unbc.ca; 6 Center for Great Expectations, Somerset, NJ 08873, USA; 7 Department of Pediatrics, Faculty of Medicine, McGill University, Montreal, QC H3A 0G4, Canada

**Keywords:** rejection sensitivity, work perceptions, client perceptions, trauma-informed care, attachment, regulation and competency (ARC)

## Abstract

Despite practice models of trauma-informed care (TIC) emphasizing relational engagement and emotional attunement as critical to service delivery, the role of individual dispositions in shaping staff perceptions and behavior remains underexplored. This study examined how rejection sensitivity, a construct grounded in attachment theory, defined as a dispositional tendency to anxiously expect and overreact to perceived rejection, may influence staff perceptions of their roles and client relationships in residential mental health agencies implementing TIC. We further explored whether individual and organizational factors, including job satisfaction, prior trauma training, perceived isolation at work, and trauma-related knowledge, contribute to these associations. Regression analyses were conducted on survey data from 155 frontline staff across three agencies testing the associations between rejection sensitivity and two relational outcomes: perceptions of work and of clients. Higher rejection sensitivity was significantly associated with more disengaged perceptions of work and less empathic views of clients, even after controlling for demographic and contextual organizational variables. Job satisfaction and trauma knowledge emerged as domain-specific protective factors, reducing the negative impact of rejection sensitivity. The findings underscore the importance of addressing staff relational dispositions to sustain effective TIC implementation. Enhancing job satisfaction and trauma knowledge may help support staff engagement in trauma-informed practice.

## 1. Introduction

Trauma-informed care (TIC) refers to an overall framework for models of human service delivery that acknowledges the widespread impact of trauma and emphasizes environments that promote safety, trust, and healing (Hanson et al., 2018). Especially in mental health and child welfare settings, staff are expected to navigate emotionally demanding relationships with clients which require not only clinical skills but also relational sensitivity and attunement (Bosk et al., 2020). Therefore, the quality of staff–client relationships plays a crucial role in the successful implementation of TIC. Beyond technical compliance, effective trauma-informed practice depends on the emotional presence, mutual respect, and empathic responsiveness of frontline workers (Goldstein et al., 2024; Sweeney et al., 2018; Substance Abuse and Mental Health Services Administration, 2014).

Relational stability within TIC implementation requires staff to manage intense emotional labor, adapt to complex client needs, and sustain compassionate engagement under stress (Bosk et al., 2020). This load may be particularly heightened in work contexts such as these, where work goals and outcomes can be long-term, hard to define, and difficult to measure. These demands can create significant workload and emotional strain, especially when organizational support is lacking or when staff bring dispositional or trauma-related vulnerabilities into their roles. This relational burden not only affects staff well-being but may also undermine the effectiveness of TIC implementation (Sweeney et al., 2018; Knight, 2015).

These relational and emotional demands highlight a central challenge of TIC. The framework emphasizes relational safety and support, yet the conditions required to sustain these practices are often difficult to achieve in everyday organizational life. Despite its promise, implementing TIC remains challenging. Agencies often struggle to translate TIC principles into daily practice because staff receive inconsistent training, limited reflective supervision, and work within organizational structures that do not reliably support collaborative decision making (Huo et al., 2023; Mahon, 2022). Leadership priorities may not align with TIC values, creating uncertainty about how to apply trauma-informed principles in fast-paced or crisis-driven health care environments (Goldstein et al., 2024). These conditions make it difficult for frontline staff to sustain relational consistency and emotional balance.

Recent scholarship has highlighted TIC as a framework for addressing staff burnout (Elisseou, 2023) and promoting organizational resilience and leadership-level change (Elisseou et al., 2024). These efforts underscore the importance of attending to both the relational experiences of frontline staff and the broader organizational conditions that shape the implementation of trauma-informed principles.

While much of the literature on TIC has focused on organizational structures and client outcomes, a growing body of research has begun to explore how frontline staff experience and interpret their roles in emotionally demanding environments (Bosk et al., 2020). These studies often highlight the importance of job satisfaction and organizational alignment in supporting staff well-being. However, much less is known about how dispositional factors such as relational sensitivities and prior emotional experiences interact with these workplace dynamics.

This study builds on and extends this perspective by examining how rejection sensitivity, conceptualized as a dispositional relational vulnerability, shapes staff perceptions of both their work and their clients within mental health agencies. While job satisfaction is known to influence how staff relate to their professional roles, it may not fully account for the emotional and interpersonal dynamics that rejection sensitivity helps to explain. In doing so, it contributes to a more nuanced understanding of job satisfaction, viewing it not only as a product of organizational conditions but also as a relational experience shaped by individual traits.

### 1.1. Rejection Sensitivity as a Relational Risk Factor

Rejection sensitivity, a concept grounded in attachment theory, is a dispositional tendency to anxiously expect, readily perceive, and strongly react to perceived rejection in social interactions (Downey & Feldman, 1996). Individuals high in rejection sensitivity tend to interpret ambiguous or neutral cues as rejecting. This often triggers defensive or avoidant responses that undermine relationship quality (Romero-Canyas et al., 2010; Stafford, 2007). This pattern reflects a cognitive-affective-behavioral defensive system that sensitizes individuals to potential social threat and shapes their interpersonal behavior (Pietrzak et al., 2005).

Empirical studies have consistently linked rejection sensitivity to difficulties in maintaining positive interpersonal relationships, including increased conflict, withdrawal, and self-fulfilling patterns of rejection that undermine relationship quality (London et al., 2007; Downey et al., 1998). In women, rejection sensitivity has been associated with heightened hostility and emotional withdrawal in response to perceived rejection (Ayduk et al., 1999), as well as with greater depressive symptoms and difficulties in regulating emotional responses (Ayduk et al., 2001). Research also suggests that rejection sensitivity disrupts attentional control in the presence of social threat cues, which may impair effective engagement in social situations (Berenson et al., 2009).

Beyond general interpersonal contexts, rejection sensitivity has also been examined in organizational settings. Among tenured and tenure-track business school faculty, higher rejection sensitivity has been linked to lower organizational commitment, diminished collegial engagement, and greater intent to leave the institution (Day & Porter, 2018), suggesting that relational vulnerabilities may shape how individuals navigate interpersonal demands in professional environments. Outside of academic workplaces, however, empirical research on rejection sensitivity in broader occupational settings remains limited. This is notable given that many human-service environments place particularly high relational and emotional demands on staff.

Within TIC settings, emerging evidence has begun to document the implications of rejection sensitivity for frontline staff. Bosk et al. (2020) found that staff with higher rejection sensitivity were less likely to endorse TIC principles and more resistant to adopting trauma-informed interventions. Building on their work, Hardan et al. (2023) demonstrated that rejection sensitivity was negatively associated with organizational attachment and positively associated with turnover intentions among mental health agency staff. Together, these findings suggest that personal relational dispositions, such as rejection sensitivity, may interact with the high-level relational and organizational demands of TIC in ways that could shape staff engagement and the quality of care delivered.

### 1.2. Staff Perceptions of Work

The relational nature of staff perceptions of work lies in how workers integrate their sense of self with their professional identity and the meaning they ascribe to their roles within the organization. Positive perceptions of work, characterized by a sense of alignment, efficacy, and respect, have been linked to greater job satisfaction, organizational commitment, and resilience in the face of stress (T. W. Hales et al., 2017; Amateau et al., 2023). While related to job satisfaction, such perceptions reflect not just evaluative satisfaction with job conditions, but also relational and emotional meaning-making around one’s role, especially meaningful in TIC settings.

Favorable attitudes toward TIC similarly appear to protect staff from stress and disengagement, underscoring the role of professional alignment with TIC principles in sustaining well-being (Wholeben et al., 2023; Minne & Gorelik, 2022). However, when staff perceive their roles as unsupported, inequitable, or misaligned with their values, they may experience diminished efficacy and disengagement from trauma-informed practice (Stevens et al., 2019; Kirst et al., 2017). In this way, staff perceptions of work reflect both how they interpret their responsibilities and how they see their own value within care relationships, shaping their ability to sustain trauma-informed principles in practice.

While organizational support and professional identity have been recognized as important for sustaining trauma-informed care, much of the empirical research has focused on structural factors such as workload, leadership, and organizational climate, as well as on staff outcomes like burnout and turnover (Bryson et al., 2017; Amateau et al., 2023; Fernández et al., 2023). These studies highlight the significance of organizational environments but have given less attention to the ways individual relational orientations toward work, including how staff understand their roles, perceive their value, and align themselves with TIC principles, influence engagement in practice.

These dynamics are particularly evident in residential mental health settings, where staff face intense emotional demands and prolonged exposure to client crises. In such environments, they must navigate challenging interactions with trauma-exposed clients while sustaining a sense of professional purpose and organizational alignment despite limited resources and high stress (Bosk et al., 2020). Addressing this gap is therefore critical for understanding how to support staff in maintaining the relational ethos of trauma-informed care.

### 1.3. Staff Perceptions of Clients

Staff perceptions of clients are a core relational dimension of TIC, shaping how frontline workers interpret client behavior and how they see their own role within these interactions. When staff perceive clients as deserving of care and capable of growth, they are more likely to respond with empathy, attunement, and patience, fostering a therapeutic environment aligned with TIC principles (Kirst et al., 2017; Mefodeva et al., 2023). Alternatively, perceiving clients as resistant, hostile, or unworthy of support may prompt staff to disengage emotionally, respond punitively, and compromise the relational climate required for healing (Amateau et al., 2023; Knight, 2015). These perceptions not only influence the immediate quality of staff–client relationships but also reflect deeper attitudes that can sustain or erode the relational ethos of TIC implementation.

Beyond shaping external interactions, staff perceptions of clients also influence how workers understand their role within therapeutic relationships and their confidence in navigating challenging encounters (Knight, 2015; Sweeney et al., 2018). When staff feel disconnected from or misaligned with their clients, they may experience diminished professional efficacy and reduced commitment to trauma-informed principles, partly due to decreased confidence and misaligned beliefs about trauma (Sundborg, 2019). Thus, perceptions of clients function as both reflections and drivers of staff resilience and relational engagement in practice.

Existing research in TIC settings has largely focused on organizational climate, system-level supports, and staff outcomes such as burnout, secondary traumatic stress, and job satisfaction (Sweeney et al., 2018; Bryson et al., 2017). For example, the Trauma-Informed Climate Scale (TICS-10) assesses perceptions of safety, trust, and empowerment at the organizational level, reflecting the field’s emphasis on systemic factors (T. Hales et al., 2019). However, much less is known about how individual dispositions shape staff perceptions of clients and their interactions.

This gap is especially relevant in residential mental health settings, where staff work closely with families in which children have experienced trauma as a result of parental substance use, maltreatment, neglect, or inconsistent caregiving (Bosk et al., 2020). These parents often display hostility, emotional instability, role reversal, and difficulty in caregiving, which can create intense relational strain and test the limits of staff empathy and emotional resilience. Understanding how staff perceive and relate to clients in such challenging contexts is therefore critical for supporting their ability to sustain TIC.

Understanding how staff perceive their clients is essential not only for fostering relational alignment but also for sustaining TIC under the emotional demands of residential settings. While both perceptions of work and perceptions of clients reflect staff’s relational orientation, they capture distinct aspects of engagement. Perceptions of work concern how staff view their professional roles and organizational fit, whereas perceptions of clients focus on how staff interpret client behaviors and how those interpretations shape therapeutic relationships and their own professional identity.

Perceived isolation at work describes employees’ subjective sense of being excluded from social connections and detached from organizational goals, even when others are physically present and formal support structures exist (Marshall et al., 2007; Sahai et al., 2020). It reflects both social isolation, which refers to limited relationships with peers, and organizational isolation, which refers to a sense of being removed from the collective mission. Staff may technically have access to others yet still feel excluded from meaningful collaboration, carrying responsibilities in a way that feels solitary and unrecognized (D’Oliveira & Persico, 2023).

This phenomenon is particularly relevant in TIC settings, which are characterized by high emotional workload, ambiguous outcomes, and progress that is difficult to measure (Meese et al., 2024; Knight, 2015; Sweeney et al., 2018). The difficulty of evaluating progress, in particular, can be understood as a reflection of what Preston (2015) describes as “indeterminate organizational technologies,” or work environments where action-outcome relations are difficult to predict or evaluate, making it hard for staff to determine whether their actions are effective and valued. In such contexts, perceived job autonomy alone may not reduce stress; its effectiveness appears to hinge on the availability of instrumental feedback and role clarity that support this interpretive process (Preston, 2015).

### 1.4. Perceived Isolation at Work

Perceived isolation may be one manifestation of this broader operational uncertainty: even when structural supports are formally in place, the absence of timely, relational, or goal-related feedback can leave staff feeling disconnected. As a result, resilience may depend on feeling “in the fight” alongside a supportive team (Bosk et al., 2020), rather than formal support structures, and perceived isolation risks depriving staff of this sense of shared purpose. Conversely, feeling supported and aligned with organizational goals has been associated with greater commitment to TIC, which may help staff sustain empathy and engagement even under stress (Sundborg, 2019).

Emerging evidence suggests that perceived isolation can impede TIC implementation. Berg-Poppe et al. (2022) found that even after TIC training improved knowledge and self-efficacy, staff still described their work as fragmented and siloed due to institutional and disciplinary barriers. Similarly, Cerny et al. (2023) reported that staff often felt disconnected, unable to collaborate, and constrained by rigid schedules and high caseloads. These findings echo earlier theoretical claims that organizational supports like autonomy require interpretive resources like feedback and clarity to be effective in complex, human-centered systems (Preston, 2015). Recognizing and measuring perceived isolation is therefore crucial for understanding not just whether support systems exist, but whether staff truly experience a sense of connection and shared purpose in their work. By capturing this subjective experience, the concept could help to assess the effectiveness of organizational supports and identify areas where formal structures fail to foster meaningful collaboration, even when they are present.

Taken together, the key constructs examined in this study offer a relational framework for understanding how frontline staff experience both strain and support in trauma-informed workplaces. This integrated perspective makes it possible to examine how dispositional vulnerabilities interact with organizational and interpersonal conditions to shape key outcomes such as emotional well-being, professional alignment, and engagement with clients. While job satisfaction remains a widely recognized indicator of staff well-being, this study complements existing approaches by introducing a relational lens that considers how individual vulnerabilities, such as rejection sensitivity, may shape how staff interpret and respond to emotionally demanding environments.

TIC implementation places substantial relational and emotional demands on frontline staff in human service organizations, making their well-being a core determinant of service quality and sustainability. Trauma-informed settings rely on staff who can maintain emotional balance, feel supported in their roles, and remain connected to the relational goals of the work. Examining how rejection sensitivity—a dispositional factor rooted in attachment theory—shapes staff perceptions of their work and their clients can deepen our understanding of employee well-being and job satisfaction, both of which are central to sustaining a healthy work environment.

This study therefore contributes to this wider conversation by examining how individual relational tendencies interact with organizational conditions to influence how staff view their roles and their clients. In doing so, it highlights that a healthy work environment depends not only on structural supports but also on the personal relational patterns that staff bring into their work.

In this study, building on the work of Bosk et al. (2020), who identified rejection sensitivity as a key factor in staff endorsement of TIC and intent to turnover, we expand the scope of inquiry to consider how rejection sensitivity may influence frontline workers’ perceptions of their own professional roles and their relational stance toward clients. This research complements the findings of Hardan et al. (2023), who examined organizational outcomes, by focusing instead on relational outcomes central to trauma-informed practice.

Drawing from the ARTIC scale, we constructed two composite variables that reflect key relational dimensions of TIC practice: (1) *Perceptions of work*, which capture the extent to which staff experience their roles in emotionally open and responsive terms; and (2) *Perceptions of clients*, which reflect attitudes toward clients’ intentions, needs, and relational behavior.

The primary aim of this study is to assess whether rejection sensitivity predicts more negative staff attitudes in these two domains. We also explore whether these associations are moderated by additional factors such as prior trauma training, perceived workplace isolation, job satisfaction, and attachment security.

Based on existing literature, we propose the following hypotheses:

**Hypotheses** **1 (H1).** *Rejection sensitivity will be negatively associated with staff perceptions of work, such that higher rejection sensitivity predicts more disengaged or emotionally withdrawn attitudes toward one’s professional role.*

**Hypotheses** **2 (H2).** *Rejection sensitivity will be negatively associated with staff perceptions of clients, such that staff higher in rejection sensitivity will report less empathetic and more rigid relational attitudes toward clients.*

**Hypotheses** **3 (H3).** *The associations proposed in H1 and H2 will be moderated by individual and organizational factors. Specifically: (a) Secure attachment, job satisfaction, and prior trauma training are expected to buffer the negative effects of rejection sensitivity on staff perceptions; (b) Isolated practice and lower levels of trauma-related knowledge are expected to exacerbate the negative associations.*

These hypotheses aim to advance our understanding of how dispositional relational vulnerabilities shape the implementation of trauma-informed principles in organizational contexts that demand high emotional labor and relational flexibility.

## 2. Materials and Methods

### 2.1. Data Source and Sample

This study carried out a secondary data analysis using survey data collected from frontline staff, supervisors, and administrators across three mental health agencies in the northeastern United States, all of which were implementing the Attachment, Regulation, and Competency (ARC) model of TIC. The original data were gathered between 2017 and 2022 as part of a larger implementation study. For details on the data collection procedures, sampling frame, and survey instrumentation, see Bosk et al. (2020). The study leads developed strong relationships with the participating agencies and their staff. Recruitment involved direct outreach to all frontline staff with agency supported time for data completion. Participation was voluntary but highly encouraged, and staff were reassured that responses were confidential from the employer. Given this near-complete coverage, the risk of self-selection bias was minimal.

Due to missing data on some survey items, the available sample size varied across the dataset, with complete responses ranging from 180 to 155 cases depending on the variables included. To ensure consistency and comparability across regression models, we restricted our analytic sample to the 155 participants who provided complete data on all variables relevant to the present study. Descriptive results are presented in Table 1.

Race was coded as a binary variable (Non-White = 1, White and Non-Hispanic = 0) in the original dataset, following the structure used in Hardan et al. (2023), and was retained as such for the current analysis. Educational attainment ranged from high school to doctoral degree, with the majority of respondents (54.2%) having completed a master’s degree. Staff position categories were based on self-report and allowed for multiple selections, resulting in total percentages exceeding 100%. These categories are presented descriptively and were not included in the current analysis.

### 2.2. Measures

#### 2.2.1. Independent Variable

Rejection sensitivity was measured using the Adult Rejection Sensitivity Questionnaire (Berenson et al., 2009), an 18-item instrument that presents hypothetical interpersonal scenarios adapted from Downey and Feldman’s (1996) original Rejection Sensitivity Questionnaire. Responses are scored on 6-point Likert scales. Composite scores reflect the product of concern and expectation across all items, with higher scores indicating greater rejection sensitivity. In the Bosk et al. (2020) study, the measure demonstrated good internal consistency (Cronbach’s α = 0.84). In the present analytic sample, Cronbach’s alpha was 0.83.

#### 2.2.2. Dependent Variables

Perceptions of work were assessed using a 10-item composite scale drawn from the attitudes related to trauma-informed care (ARTIC) measure in the original dataset, selected to capture staff members’ attitudes toward their own professional roles. The ARTIC was originally developed and validated by Baker et al. (2016), who reported strong internal consistency. In Bosk et al. (2020), the overall ARTIC score demonstrated excellent reliability (Cronbach’s α = 0.95). Items in the present study followed a bipolar format in which respondents indicated their position between two contrasting statements. For example: “It’s best not to tell others if I have strong feelings about the work because they will think I am not cut out for this job” versus “It’s best if I talk with others about my strong feelings about the work so I don’t have to hold it alone.” Responses were rated on a 7-point scale, with higher scores reflecting more emotionally open, relationally engaged, and flexible attitudes. Because the present analysis used a concept-guided subset of ARTIC items, this composite does not correspond to an official ARTIC subscale with independent validation. The scale’s internal consistency was acceptable (Cronbach’s alpha = 0.78).

Perceptions of clients were assessed using a 13-item composite scale drawn from the ARTIC measure in the original dataset, following the same construction approach as the *Perceptions of work* variable. The ARTIC has demonstrated excellent internal consistency across studies (Baker et al., 2016; Bosk et al., 2020). The items reflected staff attitudes toward clients and the relational strategies used in engagement. Each item contrasted two opposing statements, and participants rated their position along a 7-point spectrum. For example, one item contrasts “rules and consequences are the best approach when working with people with trauma histories” with “focusing on developing healthy, healing relationships is the best approach when working with people with trauma histories.” Higher scores reflect more empathetic, open, and flexible attitudes toward clients, while lower scores indicate more rigid, rule-based, or depersonalized perspectives. The scale demonstrated strong internal consistency (Cronbach’s alpha = 0.87).

#### 2.2.3. Covariates

Secure attachment was represented by a pre-existing dichotomous variable in the dataset, based on which of four statements participants identified as most reflective of their views on emotional relationships. The respondents who selected the statement reflecting ease with emotional closeness, mutual dependence, and low anxiety about rejection were coded as securely attached (1), while all others—indicating avoidant, anxious, or ambivalent tendencies—were coded as insecurely attached (0). This variable served as a proxy for general attachment security. Prior trauma training was a dichotomous variable based on participants’ self-report of whether they had received any formal training in TIC.

Perceived isolation at work was operationalized using an 11-item composite scale constructed from two domains in the original dataset, *Feelings about Supervisor* and *Support System*, which were originally examined as separate constructs in Bosk et al. (2020). This measure was designed to capture staff members’ subjective sense of relational and professional isolation, reflecting a lack of meaningful support and collaboration at work. The conceptualization of this construct was informed by Thornton et al. (2025), Cerny et al. (2023) and Berg-Poppe et al. (2022), who highlighted the relational risks of workplace isolation in trauma-informed care contexts. Guided by this framework, we identified 11 conceptually relevant items that reflect staff members’ perceived lack of relational and supervisory support in the workplace. Each item was rated on a 4-point agreement scale, ranging from 1 = Strongly Agree to 4 = Strongly Disagree. Higher total scores indicate greater perceived professional and relational isolation in workplace. A sample item includes: “Do you feel that you get the support you need from your work supervisors in order to cope with challenges?” This composite has not been externally validated as an independent scale, but demonstrated excellent internal consistency in the present sample (Cronbach’s alpha = 0.92).

Knowledge about violence and trauma was represented by a composite variable included in the original dataset, consisting of 25 items assessing participants’ self-reported knowledge related to the causes, effects, and clinical implications of violence and trauma. Adapted from Postmus et al. (2011), the measure demonstrated excellent reliability in Bosk et al. (2020) (Cronbach’s α = 0.95). Higher scores indicate greater perceived understanding of trauma-related concepts and their relevance to clinical practice. Job satisfaction, also drawn from the Bosk et al. (2020) dataset, was represented by a composite variable of 23 items capturing multiple dimensions of workplace experience, including perceptions of role clarity, autonomy, recognition, collegiality, supervisory support, communication, workload manageability, and alignment with organizational values. This composite has not been externally validated as an independent scale. Higher scores reflect greater overall satisfaction with one’s professional environment. In the current analytic sample, both scales demonstrated excellent internal consistency (Cronbach’s alpha = 0.95 and 0.90, respectively).

#### 2.2.4. Control Variables

The following demographic characteristics were included as control variables in all regression models: sex, race, education level, and annual income (following the original dataset structure from Hardan et al., 2023). These variables were selected to account for sociodemographic influences on staff perceptions.

### 2.3. Analytic Approach

We conducted ordinary least squares (OLS) regression analyses to examine the associations between rejection sensitivity and two outcome variables: perceptions of work and perceptions of clients. Separate models were estimated for each dependent variable (see Table 2 and Table 3). All analyses were conducted using IBM SPSS Statistics, Version 29.0.2.0.

To investigate the role of rejection sensitivity in shaping staff attitudes toward their work and their interactions with clients, we estimated two sets of layered OLS regression models, one for each dependent variable. The same model structure was applied across both outcomes.

In Model 1, rejection sensitivity was entered as the sole predictor. Model 2 introduced secure attachment as a theoretically relevant psychosocial variable. However, as this predictor was not significant, it was not retained in subsequent models. Model 3 added demographic controls, including sex, race, education level, and annual income. Model 4 included prior trauma training and isolated practice. Model 5 introduced knowledge about violence and trauma, and Model 6 added job satisfaction as the final covariate.

## 3. Results

### 3.1. Perceptions of Work

As shown in Table 2, rejection sensitivity significantly predicted more negative perceptions of work in Model 1 (β = −3.51, *p* < 0.001). This association remained stable in Model 2, where secure attachment was added but found to be insignificant.

Model 3 introduced demographic variables, none of which emerged as significant predictors. Rejection sensitivity remained a significant predictor (β = −3.48, *p* < 0.001). In Model 4, prior trauma training and isolated practice were added. Prior trauma training had a negative but insignificant association. Isolated practice was significantly and negatively associated with perceptions of work (β = −0.21, *p* < 0.05). The effect of rejection sensitivity remained strong and significant (β = −3.26, *p* < 0.001).

In Model 5, knowledge about violence and trauma was found to be a significant positive predictor of perceptions of work (β = 3.87, *p* < 0.05). In Model 6, job satisfaction stood out as the strongest predictor (β = 4.41, *p* < 0.01), while rejection sensitivity remained significant (β = −2.23, *p* < 0.05), though its influence was further reduced. The final model explained 24% of the variance in perceptions of work.

### 3.2. Perceptions About Clients

Table 3 presents the regression results predicting staff perceptions of clients. In Model 1, rejection sensitivity significantly predicted more negative perceptions of clients (β = −3.41, *p* < 0.01). This association remained robust in Model 2, where secure attachment was added but was not statistically significant.

Model 3 introduced demographic covariates. Educational level was identified as a significant positive predictor (β = 1.90, *p* < 0.01), while the effect of rejection sensitivity remained significant (β = −3.38, *p* < 0.01). Model 4 added prior trauma training and isolated practice. Prior trauma training showed a significant negative association with perceptions of clients (β = −3.41, *p* < 0.05), while isolated practice was insignificant. The effect of rejection sensitivity continued to be statistically significant (β = −3.27, *p* < 0.01).

In Model 5, knowledge about violence and trauma was added and found to be significantly and positively associated with perceptions of clients (β = 5.03, *p* < 0.05). Rejection sensitivity remained significant (β = −2.45, *p* < 0.05). In the final model, knowledge about violence and trauma remained a significant positive predictor of perceptions of clients (β = 4.66, *p* < 0.05). Job satisfaction, which was introduced in this step, was positively associated but not statistically significant. Rejection sensitivity continued to be a significant negative predictor (β = −2.29, *p* < 0.05). The full model explained 24% of the variance in perceptions of clients.

Multicollinearity was assessed for the final model 6 in both Table 2 and Table 3 using tolerance and variance inflation factor (VIF) values. All tolerance values were ≥0.2 and all VIFs were <5 with most close to 1, indicating that multicollinearity was not a concern. To assess whether the results were driven by highly influential observations, we examined Cook’s distance values. Using the common 4/n criterion (4/155 ~0.026), cases with Cook’s D > 0.026 were excluded and the final model 6 in both Table 2 and Table 3 were re-estimated. All predictors that were significant in the full sample remained significant, and the effect of rejection sensitivity was slightly stronger. These results indicate that the findings are robust and not unduly influenced by a small number of cases.

## 4. Discussion

### 4.1. Rejection Sensitivity Across Relational Domains

This study examined how rejection sensitivity shapes staff perceptions of their work and their clients within trauma-informed care (TIC) settings. Across both domains, rejection sensitivity consistently remained as a significant negative predictor, even after accounting for demographic characteristics, individual and organizational factors. These findings align with prior research identifying rejection sensitivity as a dispositional vulnerability that undermines organizational attachment (Hardan et al., 2023).

However, the current study extends these insights by demonstrating that rejection sensitivity is not limited to shaping organizational attitudes but also shapes relational dynamics at the core of trauma-informed practice. That is, rejection sensitivity appears to affect how staff interpret the emotional demands of their roles as well as how they understand and relate to clients, which are two domains central to the relational orientation of TIC. This cross-domain consistency suggests a potential pattern in which rejection sensitivity contributes to a general erosion of relational alignment within care environments, highlighting the need for trauma-informed frameworks to address not only clients’ trauma histories but also dispositional relational risk factors affecting staff functioning.

This highlights the importance of understanding not only what supports relational alignment, but also what undermines it, suggesting that dispositional risk factors may warrant as much attention as promotive factors such as job satisfaction.

### 4.2. Domain Specific Protective Factors

Although rejection sensitivity was the most consistent predictor across models, the study also revealed distinct domain-specific protective factors. Job satisfaction was the most robust predictor of work perceptions, suggesting that staff who feel supported, recognized, and professionally aligned may be buffered against the disengaging effects of rejection sensitivity. By contrast, perceptions of clients were most strongly predicted by knowledge about violence and trauma. This distinction aligns with the relational challenges highlighted earlier in residential mental health settings, where staff are called to sustain professional engagement and empathy despite difficult interactions, limited resources, and ambiguous outcomes.

These findings suggest that staff draw on different resources to navigate relational strain in these two domains. Job satisfaction may function as an organizational buffer, enhancing role stability and reinforcing staff’s sense of professional alignment and self-efficacy in their roles, while knowledge about violence and trauma may serve as a relational buffer, providing cognitive frameworks that help staff depersonalize client behavior and maintain empathetic stances, even when clients pull away emotionally or struggle to manage their feelings. This differentiation underscores the importance of tailoring organizational supports: enhancing job satisfaction may be particularly effective for maintaining professional engagement, while promoting trauma knowledge may be critical for sustaining empathic and constructive client relationships.

The distinct pathways through which job satisfaction and trauma knowledge appear to operate raise an important theoretical question: are such protective resources universally effective, or do they depend on specific contextual conditions to buffer against the relational strain that rejection sensitivity entails? Prior research suggests that structural supports may falter when staff lack the interpretive resources needed to understand the impact of their work (Preston, 2015).

While the present study did not examine autonomy or role clarity directly, it contributes to this line of inquiry by highlighting how different forms of support may function differently across relational domains. Understanding the conditions under which protective resources succeed or fall short remains critical for designing trauma-informed systems that are both relationally responsive and structurally attuned. These findings add nuance to existing literature on employee well-being by showing that job satisfaction may be necessary but not sufficient for relational alignment in TIC contexts. Dispositional vulnerabilities such as rejection sensitivity may help explain why some staff continue to experience relational strain despite high levels of job satisfaction.

### 4.3. Mechanism Hypothesis: The Role of Education and Trauma Knowledge

Among demographic variables, only education level was consistently associated with more positive perceptions of clients. Supplementary correlation analysis further indicated a significant positive association between education level and knowledge about violence and trauma (r = 0.40, *p* < 0.001), suggesting a possible pathway through which education fosters relational capacities. While causal inferences cannot be drawn, one plausible mechanism is that staff with higher educational attainment may possess stronger learning and integrative capacities, which enable them to acquire, retain, and apply trauma-related knowledge throughout their careers.

Importantly, this trauma-related understanding may not stem solely from having previously received formal education or training in trauma frameworks. Rather, it may reflect an ongoing ability to integrate such knowledge into day-to-day practice over time, deepening their capacity to engage constructively with clients.

This mechanism seems particularly relevant in residential mental health settings, where staff often simultaneously support children while engaging with their parents, often under emotionally demanding circumstances. In such contexts, trauma knowledge may help staff sustain empathy and relational engagement by providing frameworks to reinterpret parental behaviors, including withdrawal, affective dysregulation, and even signs of parenting struggle or harshness, not as intentional harm but as expressions shaped by their own histories of trauma, pain, and loss. This understanding allows staff to maintain a therapeutic stance even when witnessing the impact of these behaviors on children.

This proposed pathway remains hypothetical in the present study but points to a valuable direction for future research, which is to examine how education and trauma knowledge work together to foster relational alignment in practice. It highlights the potential of trauma-related knowledge not merely as content, but as a clinical tool that helps staff interpret emotionally charged situations more constructively and respond with sustained empathy and professionalism.

### 4.4. Implications, Limitations and Future Research

The findings of this study reinforce the interplay between employee well-being and job satisfaction and have important implications for understanding how healthy trauma-informed work environments can support those processes. These two elements are increasingly recognized as core conditions for sustaining trauma-informed practice. The results show that both organizational factors and individual relational tendencies contribute to shaping how staff experience their roles and their relationships with clients, underscoring the interconnected conditions that enable a stable and effective workforce. First, rejection sensitivity appears to operate as a dispositional risk factor that cuts across both staff perceptions of their professional roles and their relationships with clients. This suggests that efforts to foster relational alignment in trauma-informed settings must go beyond external supports and consider the personal emotional patterns that shape how staff relate to their work and clients. These results suggest that strengthening both individual capacities and organizational resources may enhance employee well-being and job satisfaction in trauma-informed systems, consistent with the broader goals of promoting healthy, meaningful and sustainable workplaces. Organizations can support this goal by incorporating supervision and team routines that allow staff to discuss interactions that feel tense or ambiguous. These practices can reduce misinterpretations and strengthen shared understanding, and may be especially helpful for staff who have higher level of rejection sensitivity.

Second, the identification of domain-specific protective factors points to the need for differentiated intervention strategies: enhancing job satisfaction may strengthen professional engagement, while expanding trauma-related knowledge may be especially important for supporting empathy and emotional regulation in worker-client interactions. For organizations, these findings highlight the importance of treating job satisfaction as a central indicator when planning and reviewing trauma-informed initiatives, rather than as a secondary outcome. Attending to staff satisfaction and considering it explicitly in planning and evaluation may be one way to support sustained engagement in emotionally demanding roles. In turn, the association between trauma knowledge and staff perceptions of clients suggests that trauma knowledge may be most useful when they function as ongoing interpretive tools. When brought into routine supervision and case discussions, trauma knowledge can help staff make sense of challenging interactions in ways that support more empathic and regulated engagement in daily practice.

Third, the observed link between educational attainment and trauma knowledge highlights a possible long-term pathway through which staff develop the relational competencies needed for trauma-informed practice. Organizations may benefit from supporting continuous learning environments that allow for the integration of clinical knowledge into relational skill-building over time.

Finally, this study reinforces the call by Mendez et al. (2023) to further refine the ARTIC as a measurement tool in trauma-informed care. The present findings suggest that existing instruments may not fully capture the relational tendencies and emotional patterns that shape how staff engage with their work and their clients. Continued development of measures that assess these relational dimensions would strengthen future research and improve the evaluation of trauma-informed practice.

Several limitations should be acknowledged. First, the cross-sectional design of this study precludes any conclusions about causality or temporal ordering. The associations between some of these constructs are likely to be bidirectional, or transactional, over time, and longitudinal approaches would allow for more nuanced direction of effects understanding. Second, all variables were based on self-report measures, raising the possibility of common method bias. And, despite strong workplace supports for protected time for staff to complete measures and relationship building liaison efforts with the organizations, participation was voluntary, raising the potential for self-selection bias in staff participation that could influence broader generalizability. Third, as noted in the Measures section, the operationalization of perceived isolation at work in this study reflects only a limited aspect of the broader construct, capturing primarily its relational and supervisory dimensions. This restricted scope underscores the need for more comprehensive and nuanced measures of perceived isolation in future research. Fourth, the relatively small sample size limits the generalizability of the findings and constrains the statistical power for more complex modeling approaches. Finally, our study controlled for available organizational variables, but other contextual factors were not measured (e.g., agency policies, supervisory practices). Further exploration of additional organizational influences on staff experiences in TIC settings could better elucidate those interpersonal-organizational reciprocal chains of influence.

Future research could explore whether trauma-related knowledge helps explain the association between educational attainment and staff perceptions of clients. While the current study did not formally test this mechanism, the observed associations suggest a conceptual pathway worth investigating. Mediation analysis may offer one way to examine whether trauma knowledge functions as a key link between education and relational stance.

Additionally, longitudinal research could examine how rejection sensitivity, trauma knowledge, and staff perceptions evolve over time, particularly during periods of organizational change, new practice model implementation, or staff training.

Finally, using observational methods or gathering data from multiple informants could provide richer insights into how relational attitudes are enacted in daily practice, and how they interact with organizational climate and client outcomes. Future studies could benefit from refining the measurement of perceived isolation at work to better capture its multifaceted nature and to examine its potential implications for organizational supports, staff engagement and TIC implementation more fully.

Beyond these empirical priorities, researchers may also consider deeper conceptual inquiries into the personal histories that shape professionals’ engagement with TIC. At the same time, many trauma-informed professionals themselves report high levels of trauma exposure or adverse life experiences (Knight, 2015; Bride, 2007). For some, it may be the emotional challenges of TIC that make the work feel purposeful or personally meaningful. This paradox, in which histories of pain may function not only as vulnerabilities but also as sources of meaning, purpose, and resilience, deserves further exploration. Understanding how staff’s own histories shape their relational motivations may help explain divergent responses to relational strain in TIC settings.

## Figures and Tables

**Table 1:** Secondary study sample descriptives.

n = 155	Mean or % (n)	SD	Min	Max
**Organizational Attachment**	4.40	1.13	1	6
**Rejection Sensitivity**	2.56	0.71	1.17	4.11
**Intent to Turnover**	2.20	1.18	1	5
**Sex**				
Male	14.8% (23)			
Female	85.2% (132)			
**Race**				
Non-White	40% (62)			
White	60% (93)			
**Hispanic**				
Hispanic	20% (31)			
Not Hispanic	80% (124)			
**Education Level**				
Completed HS or GED	6.5% (10)			
Some College	7.1% (11)			
Completed College	23.9% (37)			
Some Masters Completed	6.5% (10)			
Masters Completed	54.2% (84)			
Completed Ph.D. or equivalent	1.9% (3)			
**Staff Position** *				
Clinician	43.9% (68)			
Program Manager	28.4% (44)			
Residential Associate	14.8% (23)			
Child Care Worker	2.6% (4)			
Supervisor	1.3% (2)			
Case Manager	1.3% (2)			
Other Support Staff	14.2% (22)			
**Annual Income**				
<USD 20,000	2.6% (4)			
USD 20,000–USD 40,000	32.9% (51)			
USD 40,000–USD 60,000	40.6% (63)			
USD 60,000–USD 80,000	14.8% (23)			
USD 80,000+	9.0% (14)			
**Trauma Knowledge**				
Prior Trauma Training	56.1% (87)			
No Prior Trauma Training	47.9% (68)			

* Staff could choose all that apply descriptive statistics.

**Table 2:** Multiple regression predicting perceptions about work (unstandardized betas, 95% CI for B in parenthesis).

Perceptions About Work						
Variable	Model 1	Model 2	Model 3	Model 4	Model 5	Model 6
Rejection Sensitivity	−3.51 ***	−3.41 ***	−3.48 ***	−3.26 ***	−2.62 **	−2.23 *
	[−5.15, −1.88]	[−5.12, −1.71]	[−5.16, −1.80]	[−4.95, −1.57]	[−4.40, −0.85]	[−3.98, −0.49]
Secure Attachment		0.54				
		[−1.94, 3.03]				
Female ^1^			−0.61	−0.76	−1.02	−1.19
			[−3.96, 2.75]	[−4.08, 2.56]	[−4.31, 2.27]	[−4.39, 2.01]
Race ^2^			−0.01	−0.08	−0.30	0.12
			[−2.43, 2.45]	[−2.33, 2.48]	[−2.71, 2.10]	[−2.24, 2.47]
Educational Level			0.63	0.60	0.25	0.30
			[−0.31, 1.57]	[−0.34, 1.53]	[−0.72, 1.23]	[−0.65, 1.25]
Annual Income ^3^			0.35	−0.08	−0.22	−0.30
			[−0.69, 1.42]	[−1.16, 1.00]	[−1.30, 0.86]	[−1.34, 0.75]
Prior Trauma Training				−1.63	−0.77	−0.60
				[−4.08, 0.82]	[−3.32, 1.78]	[−3.08, 1.88]
Isolated Practice				−0.21 *	−0.18 ^4^	0.08
				[−0.39, −0.02]	[−0.36, 0.001]	[−0.16, 0.33]
Knowledge about violence and trauma					3.87 *	2.93
					[0.25, 7.48]	[−0.63. 6.50]
Job Satisfaction						4.41 **
						[1.59, 7.23]
r^2^	0.11	0.11	0.13	0.17	0.19	0.24
N	155	155	155	155	155	155

*** *p* < 0.001 ** *p* < 0.01 * *p* < 0.05. ^1^ Reference group is male. ^2^ Reference group is White. ^3^ Increments of 20,000. ^4^ *p* = 0.052.

**Table 3:** Multiple regression predicting perceptions about clients (unstandardized betas, 95% CI for B in parenthesis).

Perceptions About Clients						
Variable	Model 1	Model 2	Model 3	Model 4	Model 5	Model 6
Rejection Sensitivity	−3.41 **	−3.32 **	−3.38 **	−3.27 **	−2.45 *	−2.29 *
	[−5.54, −1.29]	[−5.54, −1.10]	[−5.47, −1.28]	[−5.36, −1.18]	[−4.64, −0.26]	[−4.50, −0.08]
Secure Attachment		0.49				
		[−2.74, 3.73]				
Female ^1^			−1.89	−2.28	−2.62	−2.69
			[−6.07, 2.29]	[−6.39, 1.83]	[−6.69, 1.44]	[−6.76, 1.38]
Race ^2^			0.30	−0.48	−0.01	0.16
			[−2.74, 3.34]	[−2.50, 3.46]	[−2.98, 2.96]	[−2.84, 3.15]
Educational Level			1.90 **	1.78 **	1.34 *	1.35 *
			[0.73, 3.07]	[0.63, 2.94]	[0.13, 2.54]	[0.15, 2.56]
Annual Income ^3^			0.80	0.18	0.003	−0.03
			[−0.51, 2.10]	[−1.15, 1.52]	[−1.33, 1.33]	[−1.36, 1.30]
Prior Trauma Training				−3.41 *	−2.29	−2.22
				[−6.44, −0.38]	[−5.44, 0.86]	[−5.38, 0.93]
Isolated Practice				−0.22 ^4^	−0.18	−0.08
				[−0.44, 0.01]	[−0.41, 0.04]	[−0.39, 0.23]
Knowledge about violence and trauma					5.03 *	4.66 *
					[0.57, 9.50]	[0.13, 9.19]
Job Satisfaction						1.77
						[−1.82, 5.36]
r^2^	0.06	0.06	0.16	0.21	0.24	0.24
N	155	155	155	155	155	155

** *p* < 0.01 * *p* < 0.05. ^1^ Reference group is male. ^2^ Reference group is White. ^3^ Increments of 20,000. ^4^ *p* = 0.061.

## Data Availability

The deidentified data presented in this study are available on request from the corresponding authors. The data are not publicly available due to ethical restrictions related to participant confidentiality.

## Abbreviations

The following abbreviations are used in this manuscript:

ARC	Attachment, regulation and competency
TIC	Trauma-informed care
